# The adaptive significance of adult neurogenesis: an integrative approach

**DOI:** 10.3389/fnana.2013.00021

**Published:** 2013-07-16

**Authors:** Sarah Konefal, Mick Elliot, Bernard Crespi

**Affiliations:** ^1^Department of Neurology and Neurosurgery, Centre for Research in Neuroscience, The Research Institute of the McGill University Health Centre, Montreal General HospitalMontreal, QC, Canada; ^2^Department of Biological Sciences, Simon Fraser UniversityBurnaby, BC, Canada

**Keywords:** adult neurogenesis, adaptive significance, evolution, pattern separation, olfaction

## Abstract

Adult neurogenesis in mammals is predominantly restricted to two brain regions, the dentate gyrus (DG) of the hippocampus and the olfactory bulb (OB), suggesting that these two brain regions uniquely share functions that mediate its adaptive significance. Benefits of adult neurogenesis across these two regions appear to converge on increased neuronal and structural plasticity that subserves coding of novel, complex, and fine-grained information, usually with contextual components that include spatial positioning. By contrast, costs of adult neurogenesis appear to center on potential for dysregulation resulting in higher risk of brain cancer or psychological dysfunctions, but such costs have yet to be quantified directly. The three main hypotheses for the proximate functions and adaptive significance of adult neurogenesis, pattern separation, memory consolidation, and olfactory spatial, are not mutually exclusive and can be reconciled into a simple general model amenable to targeted experimental and comparative tests. Comparative analysis of brain region sizes across two major social-ecological groups of primates, gregarious (mainly diurnal haplorhines, visually-oriented, and in large social groups) and solitary (mainly noctural, territorial, and highly reliant on olfaction, as in most rodents) suggest that solitary species, but not gregarious species, show positive associations of population densities and home range sizes with sizes of both the hippocampus and OB, implicating their functions in social-territorial systems mediated by olfactory cues. Integrated analyses of the adaptive significance of adult neurogenesis will benefit from experimental studies motivated and structured by ecologically and socially relevant selective contexts.

## Discovery and analysis of adult neurogenesis

Since the original description of adult neurogenesis by Altman and Das (1965), study of this form of neurological plasticity has added a new level of complexity to our understanding of adult brain function (Ming and Song, 2011). Adult neurogenesis has since been documented in many species, including primates (Amrein et al., 2004; Barker et al., 2011; Kempermann, 2012). Recent studies have also revealed roles for neurogenic plasticity in a wide range of human physiological functions and pathological conditions (Abrous et al., 2005; Taupin, 2006).

Previous work on adult neurogenesis has focused predominantly on the cellular and molecular mechanisms that mediate its functioning. Considerably fewer studies have been directed toward understanding the adaptive significance of adult neurogenesis, with regard to cognition and behavior, in the context of variation among and within species in social and ecological traits that may have imposed selective pressures on such neuronal functions (Meltzer et al., 2005; Ming and Song, 2005; Lledo et al., 2006; Amrein et al., 2011). To understand the adaptive significance of adult neurogenesis, in terms of how and why it has evolved, three main questions can usefully be addressed:

First, what are the specific advantages of adult neurogenesis, and why, at least among mammals, is it largely restricted to the dentate gyrus (DG) and olfactory bulb (OB), in contrast to other brain regions? Presumably these two regions share some particular functional, information processing roles that benefit from neurogenetic plasticity in addition to the universal system of synaptic plasticity (Lemaire et al., 2012). However, such roles remain obscure, and indeed this question has seldom been posed in terms of specific, testable, alternative hypotheses. Are there particular forms or patterns of environmental information variability, common to the hippocampus and olfactory bulb that benefit from neuronal processing that includes neurogenesis?

Second, what are the potential costs of adult neurogenesis that may be associated with its general restriction to these two brain regions? Like all other morphological and physiological traits, the presence and intensity of adult neurogenesis is expected to involve tradeoffs between different selective pressures, and higher levels of adult neurogenesis may increase the scope for deleterious effects or dysregulation through genetic and environmental perturbations. Such potential costs have seldom been addressed, although they may impact upon the evolution of adult neurogenesis comparably in magnitude to benefits.

Third, what are the comparative correlates of adult neurogenesis, and the sizes and functions of the relevant brain regions, among sets of closely-related mammals that differ in behavioral, ecological and morphological traits that may be associated with adult neurogenesis? Most research on adult neurogenesis has involved laboratory mice or rats as model systems, experimental tasks with limited relevance to natural situations, or comparative studies that span taxa across diverse taxonomic groups. The tremendous advantages in tractability of laboratory and experimental systems are counterbalanced, as regards questions related to adaptive significance, by restrictions in the context of understanding the specific benefits and costs of adult neurogenesis among rodents living in natural situations. These restrictions can be alleviated both by increasing the ecological and behavioral validity of experimental model systems and tests, and by conducting comparative studies that use phylogenetically-distributed variation in ecology and behavior across sets of more closely-related species to evaluate hypotheses for the functions of adult neurogenesis specifically in the DG and OB.

In this article, we begin by briefly discussing adult neurogenesis in the DG and OB with reference to potentially similar functions of these regions, and the other forms of plasticity in the brain. Second, the postulated benefits and adaptive significance of neurogenic plasticity in the DG and OB will be evaluated via consideration of results from relevant computational models and ablation studies in mammals. Third, potential and documented costs associated with adult neurogenesis will be reviewed, with special attention to psychiatric conditions and brain tumor risks. Fourth, we integrate these various lines of evidence in the context of reviewing the primary models described thus far to explain the adaptive significance, and integrated proximate mechanisms, of adult neurogenesis, with a focus on why neurogenesis is restricted to the DG and OB. Finally, we present results from a comparative study of brain region sizes in haplorhine and strepsirhine primates that provides preliminary evidence regarding the behavioral and ecological correlates of joint variation in hippocampal and OB sizes and their functional importance.

## Information encoded by DG and OB

We first provide a brief overview of DG and OB circuitry, and their connections, and then discuss similarities and differences between them with regard to processing of information. This overview is important with regard to evaluating functional commonalities between the DG and OB that may help to explain the restriction of adult neurogenesis to these two regions in mammals.

**Figure 1:**
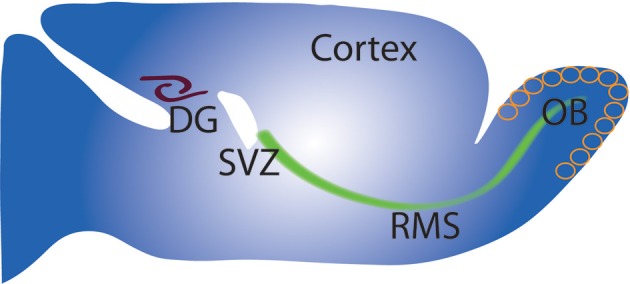
**Schematic of adult neurogenesis in the DG and OB**. Sagittal view of rodent brain (**Top left**) showing sites of neurogenesis in the SVZ/OB and SGZ/DG. Cells proliferate mainly in the SVZ, migrate along the rostral migratory stream (RMS) to the OB where (**Right**) they migrate radially and undergo differentiation and integration into OB circuit. Neuron precursors from the SGZ proliferate and migrate a short distance to the DG (left) before differentiation into granule neurons and integration into the DG circuit.

**Figure 2:**
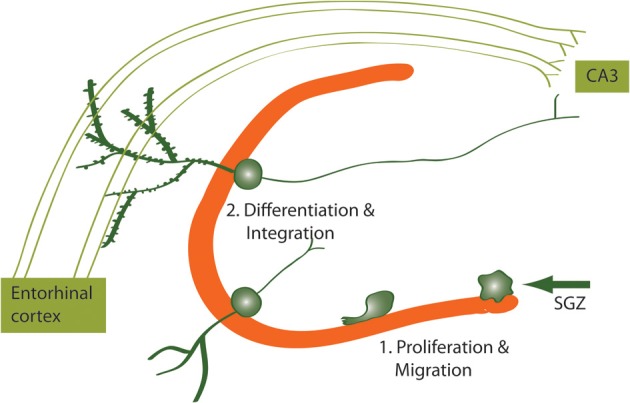
**Newborn neurons proliferate in the SGZ and migrate to the DG where they differentiate and integrate into the hippocampal circuitry**. Newborn DG neurons become excitatory granule cells receiving input from the entorhinal cortex and projecting to the CA3 subregion of the hippocampus.

Information derived from hippocampus-based learning is transformed into long-term memory storage and this function is highly conserved across species (Lindsey and Tropepe, 2006). Hippocampal dentate granule cells represent components of the synaptic circuits that receive input from the entorhinal cortex (EC) and send projections to pyramidal neurons in the CA3 region of the hippocampus (Figures 1, 2). To participate functionally in the hippocampal circuits, newly generated granule cells of the DG send axonal mossy fiber projections into mossy fibers that carry their output to hippocampal pyramidal cells (Cameron and McKay, 1999; Sohur et al., 2006). Place cells in the hippocampus encode specific spatial locations, while grid cells in the EC encode any spatial fields that are distinct from neighboring spatial fields (Hafting et al., 2005; Derdikman and Moser, 2010). Together, this circuitry is crucial for mapping position and direction in all environments. The DG receives multiple cortical and subcortical inputs, the major input being from the entorhinal cortex which processes both spatial and non-spatial information. In contrast to the many inputs to the DG, the DG only projects to the CA3 subfield of the hippocampus, thus allowing the DG to control the flow of information into the hippocampus (Figures 1, 2). The DG is an important encoding structure for spatial and associative learning and memory. In particular, the DG is able to discriminate similar spatial events from each other across all sensory inputs (Rolls and Kesner, 2006; Kesner, 2007).

**Figure 3:**
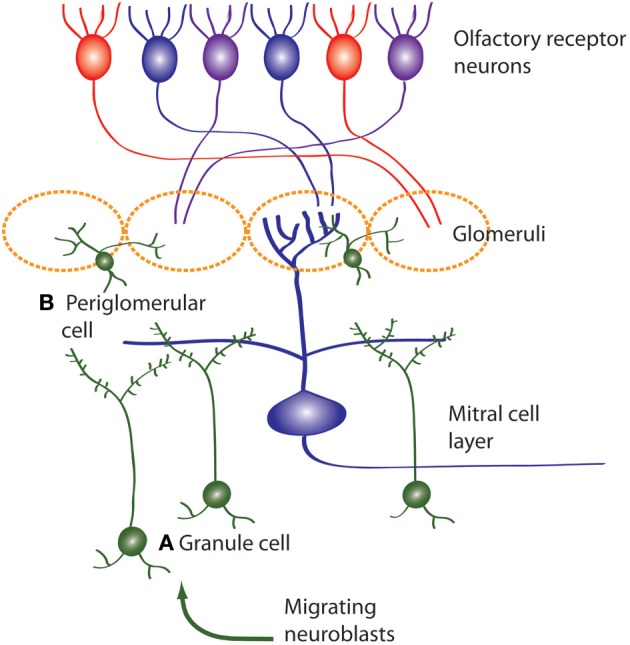
**Neuroblasts migrate to the OB from the SVZ, then differentiate and integrate into the OB circuitry**. Newborn neurons in the OB become **(A)** inhibitory granule cells and **(B)** periglomerular interneurons (also inhibitory) which both modulate mitral cell activity. Olfactory receptor neurons express one type of odor receptor and receptor neurons with the same receptor type project to the same glomerulus where they synapse onto mitral cells. Mitral cells receive information from olfactory receptor neurons and project to the olfactory cortex.

OB sensory neurons form excitatory synapses with projection neurons and with inhibitory interneurons, so that output of a given projection neuron is determined by both sensory input and activity of local inhibitory neurons that are generated throughout adult life (Lledo et al., 2008). The integration of newborn neurons in both the DG and OB follows similar temporal patterns of connectivity (Deshpande et al., 2013), although only one cell type is generated in the DG and at least two are generated in the OB (Sahay et al., 2011). The OB is connected with the hippocampus via the entorhinal cortex and amygdala, which allows for information regarding salience and emotional relevance of olfactory-related events to be conveyed for hippocampal processing and consolidation (Arisi et al., 2012; Kageyama et al., 2012) (Figure 3). This circuitry (e.g., Figure 1 in Arisi et al., 2012) may indirectly connect the effects of neurogenesis in the OB and DG, although their neurogenic niches remain physically separated.

While the DG thus appears to function predominantly as a brain structure for encoding, the vertebrate olfactory system represents an important structure for decoding an array of incoming odorant information. The degree to which neural systems specialized for complex encoding, and decoding, share functional and structural properties may provide important clues concerning the adaptive significance of adult neurogenesis, as described in more detail below. Understanding how the OB circuitry differs from the circuitry of other sensory systems will also shed light on why neurogenesis regulates olfaction but not other sensory modalities. For example, the olfactory circuit is an example of a “discrete” continuum, where the primary organization of the input field reflects discrete qualities (odorants) rather than spatial order (called a “topographic” continuum) (Luo and Flanagan, 2007; Adam and Mizrahi, 2010). Other sensory processing areas, including the visual and auditory cortex, are organized in a topographic continuum. Topographic continua may be more limited in their ability to reliably distinguish between incoming information that is very similar (Luo and Flanagan, 2007). In the context of olfaction, adult neurogenesis may thus specifically contribute to processing information that is organized by discrete qualities that can be very similar.

The OB largely consists of glomeruli. These are sites where olfactory receptor neurons synapse onto mitral neurons projecting to different cortical regions including the entorhinal cortex. In laboratory rats and mice, individual olfactory sensory neurons express only one odorant receptor out of ~1200 genes and converge onto only one or two out of ~1800 glomeruli (Buck and Axel, 1991; Mombaerts, 2004; Mori et al., 2006; Wilson and Mainen, 2006) (Figure 3). There is also very little convergence and divergence between the DG and CA3 in laboratory rats: fewer than 50 DG granule neurons synapse onto one CA3 pyramidal neuron and even fewer CA3 neurons are connected to one granule neuron (Boss et al., 1985).

The OB and hippocampus are both important for the temporary storage of information. The OB and DG also appear to process large amounts of information (Chambers et al., 2004) and use specialized coding strategies that may involve adaptation to changing environmental conditions (Cecchi et al., 2001). However, determining the functional similarities between OB and DG, in comparison to other areas without neurogenesis, has remained challenging, especially as these two brain regions differ in so many important ways.

Given that the functions of adult neurogenesis may be directly related to the generation of young neurons that differ in their patterns of synaptic plasticity (Aimone et al., 2011), it is also essential to consider the roles of synaptic plasticity itself in neuronal circuitry. Synaptic plasticity is activity-dependent change in connectivity between neurons that underlies learning, memory and many behaviors. Distinct stimuli can alter synaptic connectivity, resulting in synaptic strengthening or weakening that changes how information is encoded, represented, and retrieved. Changes in circuit function that are mediated by synaptic strengthening and weakening are not independent of adult neurogenesis, making it difficult to assess their individual contribution to DG or OB function. For instance, synaptic strengthening (called long-term potentiation, or LTP) in the DG increases the proliferation of neural precursors and enhances their survival (Bruel-Jungerman et al., 2006; Singer et al., 2011). In laboratory rats and mice, increases in LTP are observed after conditions that also stimulate adult neurogenesis (Muotri et al., 2009; Snyder et al., 2009). Importantly, adult-born dentate granule neurons express stronger synaptic plasticity than mature neurons, as indicated by their lower threshold for LTP and their higher LTP amplitude (Schmidt-Hieber et al., 2004; Ge et al., 2007). Circuit modeling studies indicate that this increased plasticity in newly integrated neurons helps to localize synaptic changes to newborn neurons, preserving information that is already represented by mature neurons for long periods of time (Deng et al., 2010). Impaired LTP in the DG results in behavioral deficits that are consistent with DG function, including fear conditioning (Saxe et al., 2006) and spatial memory (Shimazu et al., 2006). Increases in neurogenesis and LTP also improve cognition in spatial memory tasks (van Praag et al., 1999, 2005; Saab et al., 2009).

Similar to synaptic plasticity resulting from changes in synaptic weight, structural plasticity is not independent of mechanisms that regulate adult neurogenesis. Genes that regulate adult neurogenesis also modulate structural and functional properties of excitatory synapses (Pujadas et al., 2010). Structural modification of dendritic spines and axonal boutons occurs as adult-born dentate gyrus cells (DGCs) mature (Zhao et al., 2006; Toni et al., 2007, 2008). Dendritic spine density of DGCs is also regulated by LTP (Wosiski-Kuhn and Stranahan, 2012). The largest changes in dendritic spine density of newborn DG neurons occur earlier in maturation as they begin forming synapses with the existing circuitry (Kelsch et al., 2009). GABAergic synaptic signaling is important for structural maturation including dendritic branching and spine formation of adult born DG granule cells (Pallotto et al., 2012). Similar to LTP, conditions that stimulate adult neurogenesis also increase dendritic spine density in the DG (Eadie et al., 2005), indicating increased synapse formation or decreased synapse elimination. Spatial learning increases the length and branching of the DG dendritic arbors that are dependent on LTP mechanisms (Tronel et al., 2010).

Similar to findings in the DG, newborn neurons in the OB express a lower threshold for synaptic plasticity than mature neurons (Nissant et al., 2009). Behavior that correlates with enhanced synaptic plasticity of newborn neurons includes increased responsiveness to novel odors (Magavi et al., 2005), and improved olfactory discrimination learning (Mouret et al., 2008). Newborn neurons in the OB become inhibitory interneurons and granule cells that modulate the afferent activity of mitral cells (Gao and Strowbridge, 2009). The inhibitory synaptic modulation by newborn interneurons occurs at dendrodendritic synapses where the mitral cells simultaneously modulate interneuron activity. Strengthening of these synapses is likely important for promoting sparse and efficient coding of olfactory information (Rinberg et al., 2006). LTP at these synapses is required for aversive olfactory learning in a classical conditioning paradigm (Zhang et al., 2010).

Taken together, these considerations suggest the hypothesis that a primary function of neurogenesis involves the production not of new neurons *per se*, but of neurons with the synaptic-plasticity properties of relatively-young neurons (Aimone et al., 2011). This hypothesis implies that comparisons of adult OB and DG functions with neuronal functions of young neurons during neurodevelopment may lead to useful insights.

Adult born neurons in the OB undergo structural plasticity throughout their maturation and integration into OB circuits (Mizrahi, 2007). Reducing OB circuit activity lowers dendritic complexity and dendritic spine number (Dahlen et al., 2011). Olfactory network activity also modulates the integration and survival of newborn neurons. Structural plasticity of the OB has been studied in the context of social recognition and reproductive function. For instance, adult-born granule cells of the OB have fewer but more stable dendritic spines in lactating mice (Kopel et al., 2012). However, there are some instances where structural plasticity does not parallel neurogenic plasticity in the OB (Walton et al., 2012).

Although adult neurogenesis in the OB and DG differ in many ways, the organization of their circuitry and their roles in information processing exhibit several important similarities. The organization of the OB and DG circuitry both involve sparse connectivity between information input and downstream processing and storage. This network connectivity is related to the encoding/decoding roles of both the OB and DG. Lower thresholds for synaptic and structural plasticity in newborn neurons are also important for OB and DG circuit function. Looking further for parallels between the DG and OB will likely provide clues as to why neurogenic plasticity selectively regulates information processing in these brain regions.

## Potential advantages of adult neurogenesis

Adult neurogenesis has presumably evolved, and is maintained, due in part to some set of advantages which have been selected for in the context of specific forms of external, environmental information that is better-processed, stored, and deployed using the two brain systems that include neurogenesis. Comparative studies of mammalian adult neurogenesis have focused almost exclusively on the hippocampus and spatial selective pressures such as home range size, finding, for example, higher DG neuronal proliferation in rodent species with larger home ranges (Amrein et al., 2004; Barker et al., 2005; Amrein and Lipp, 2009). In contrast to rodents, relatively low rates of adult neurogenesis have been reported in primates (especially humans) and bats (Eriksson et al., 1998; Gould et al., 1999; Amrein et al., 2007). Comparative ecological or life-historical explanations of these patterns remain elusive (Amrein and Lipp, 2009). These studies are useful for developing and testing hypotheses, but comparative data are also required on OB neurogenesis, and its correlates, to determine why neurogenesis is restricted to the DG and OB. Evidence from computational modeling and ablation studies can, however, be used to evaluate hypotheses for why adult neurogenesis is largely restricted to these two brain regions, given that such studies have been conducted for both hippocampal and OB systems.

### Evidence from computational modeling

Computational studies of adult neurogenesis organize networks into three-layer systems where differential plasticity between connections can be programmed between layers, and simulated apoptotic or neurogenic events occur in the middle layer (review in Aimone and Gage, 2011). The three-layer system is similar to hippocampal structure: there is a non-neurogenic input layer (entorhinal cortex) that projects to a neurogenic middle layer (dentate gyrus) and then to a non-neurogenic output layer (mossy fibers and CA3 neurons) (Chambers et al., 2004). Mapping from the entorhinal cortex to the dentate gyrus is high-dimensional and sparse (Becker, 2005) because the DG acts to separate input patterns: it is a highly divergent structure and exposure to spatial environments is followed by DG sparse activity (Aimone et al., 2006).

The effects of neuron addition on DG encoding are increased numbers of possible distinct codes that increase hippocampal memory capacity (Becker, 2005) and reduce interference between existing memories (Wiskott et al., 2006). Neurogenic function was examined in a feed-forward network model developed by Chambers et al. (2004) to test the learning effects of neuronal turnover prior to and during learning of new alphabetic patterns. Elimination and replacement of neurons enhanced speed and accuracy of learning new information at a cost of loss of recall accuracy for old information. The loss of recall accuracy of old information is characterized as catastrophic interference by Wiskott et al. (2006). Catastrophic interference can occur if stored patterns are too similar, or because too many patterns are stored. These computational models suggest that the neurogenesis in the DG may promote the compression and ‘sparsification’ of patterns to make them more suitable for storage.

The task of the feed-forward, autoencoder network described by Wiskott et al. (2006) was to reproduce input stimulus patterns in the output layer. Two different input vectors representing two different environments were used for different input stimulus patterns. Learning ability was measured as the error between input stimulus and output reproduction when adapting to environment B after being optimized for environment A. New neurons added to the DG network helped avoid catastrophic interference by keeping old neurons adapted to earlier environments fixed, and adding new, more plastic neurons to code for aspects that are qualitatively new in the current environment. Using Hebbian learning rules, Becker (2005) proposed a coinciding role of DG neurogenesis: gradual, neurogenic changes in the internal code of the dentate layer could facilitate formation of distinct representations for highly similar experiences. This function of pattern separation was evaluated by network retrieval of training patterns for lists of unrelated items, paired associates, and related items. Memory capacity could be changed by varying numbers of training patterns to be remembered and recalled. Increasing the number of DG neurons improved recall performance for all training programs, but neuronal turnover selectively improved recall of related items that were more similar. More recent work by Clelland et al. (2009) characterizes spatial pattern separation as a process mediated by the sparse coding of the DG that is most notably affected when adult neurogenic processes are compromised.

A primary conclusion that can be drawn concerning the function of adult neurogenesis in the DG from these computational studies is that it appears to improve separation of input patterns from the entorhinal cortex and allows encoding of novel environmental stimuli while conserving memory of previously encoded information. As the entorhinal cortex and hippocampus are known to mediate directionally oriented and topographically organized neural maps of the spatial environment (Derdikman and Moser, 2010), it is reasonable to postulate that the integrated entorhinal cortex system may involve pattern separation and completion of spatially-structured information.

With regard to the roles of OB neurogenesis in learning and memory, the mathematical algorithm developed by Cecchi et al. (2001) suggests that olfactory neurogenesis maximizes discrimination of odors in a model where training is unsupervised. Basic mechanisms of the model are that neuronal incorporation proceeds at a constant unregulated rate, and that neuronal survival is modulated by activity-dependant apoptosis.

Based on Cecchi's model (Cecchi et al., 2001), the function of young neurons added to the OB in an activity-dependent fashion is to maximize the discrimination of odors in an unsupervised manner (when information is unaccompanied by any form of reinforcement). Experimental evidence in laboratory rats and mice support this model (Gheusi et al., 2000; Enwere et al., 2004; Mouret et al., 2009). The survival of the newborn neurons, and their connections to active output cells, depend on the product of the activity-dependant inputs. Young neurons have an enhanced potential for synaptic plasticity (Schmidt-Hieber et al., 2004; Lledo et al., 2006), and different maturity levels in olfactory granule cells help discriminate between odors (Gheusi et al., 2000). Olfactory discrimination is dependent on the combinatorial pattern of activation of output neurons in the OB. The activity of these output cells is regulated by inhibitory granule cells and interneurons that are generated throughout adulthood, and that have different active membrane properties that increase odor detection and discrimination (Lledo et al., 2006; Rinberg et al., 2006; Nissant et al., 2009). The process of neuronal integration may be an adaptive sensory mechanism because odors are more transient, unpredictable and complex than visual or auditory stimuli (Stockhorst and Pietrowsky, 2004), which would provide benefits to plasticity during changes in the olfactory environment. Barnes et al. (2008) describe how olfaction maintains a balance between pattern separation of overlapping input patterns and pattern completion of degraded input patterns. The decoding of spatio-temporal olfactory patterns thus clearly parallels the spatial pattern separation mediated by the DG that distinguishes variable representations of similar inputs (Clelland et al., 2009).

Based on the above experimental parameters supporting Cecchi's olfactory model, continuous granule neuron turnover in the OB may function to represent the dimensionality of sensory input of odors, which is required for odor discrimination. A more recent computational model also demonstrates that OB neurogenesis is required for fine odor discrimination, in addition to the specific detection and discrimination of novel odors (Chow et al., 2012). Thus, Chow et al. (2012) demonstrated that three properties of the OB-neurogenesis network: (1) continual addition of new inhibitory cells, (2) activity-dependent survival of new neurons, and (3) reciprocal connections with mitral cells, could generate decorrelation between representations of similar stimuli, in a similar way that DG neurogenesis mediates pattern separation via decorrelation (e.g., Aimone et al., 2011; Sahay et al., 2011; Kesner, 2013). This model provides clear evidence from theory for potential functional convergence between neurogenesis in the DG and OB.

Taken together, in the context of similarities between neurogenesis in the DG and OB, these considerations from computational modeling suggest that decoding in the OB and encoding by the DG may share functional properties that are important to the benefits of neurogenesis, especially with regard to decorrelation of similar, complex stimuli. The question then becomes how the environmental information in olfactory signals might uniquely resemble the information in spatially-structured, and other, signals that are processed in the DG.

### Evidence from ablation studies

The hippocampus responds to a wide variety of spatial inputs, including extrinsic landmarks and translational and directional movement signals (Hafting et al., 2005). Sensory information about space is differentiated by the hippocampus into a multitude of context-specific representations (Sharp, 1999), and can be retrieved from degraded versions of the original input (Hafting et al., 2005). The main functions of the hippocampus are the formation of memories and representation of space. Neurogenesis might continue throughout adulthood in brain regions that receive inputs with high statistical variability; olfactory and spatial environmental changes may thus be so varied that accommodation by synaptic plasticity alone in ineffective or inefficient (Kempermann, 2002; Nottebohm, 2002a,b). Alternatively, non-neurogenic plasticity may account for the computational benefits of adult neurogenesis in animal models. This hypothesis can be tested by targeting neurogenic plasticity and measuring cognitive and behavioral outcomes.

Studies that manipulate or target neurogenic activity in the DG in animal models show impaired formation of both short and long-term spatial memories, associations with both spatial and contextual stimuli, and cognitive flexibility when learning paradigms are altered. The ability to distinguish between similar memories is required for spatial memory formation and association with other stimuli.

Ablation studies have largely been conducted in laboratory rats and mice, and primarily involve irradiation, antimitotic agents or genetic ablation. Most studies support effects of abolished neurogenesis in either short- or long-term spatial memory (Gilbert et al., 2001; Kee et al., 2007; Dupret et al., 2008; Farioli-Vecchioli et al., 2008; Zhang et al., 2008; Deng et al., 2009; Jessberger et al., 2009; Snyder et al., 2009; Morris et al., 2012). Spatial learning and memory in laboratory rats and mice is typically investigated using different types of mazes where the animal is required to escape or locate a reward. Associative memory is another function for adult neurogenesis in the DG that is supported by ablation studies (Snyder et al., 2005; Saxe et al., 2006; Farioli-Vecchioli et al., 2008; Imayoshi et al., 2008; Hernández-Rabaza et al., 2009; Kitamura et al., 2009; Ko et al., 2009; Tronel et al., 2010). A common paradigm to examine associative memory is contextual fear conditioning where the animal is trained to associate an aversive stimulus (e.g., a foot shock) with a neutral context (e.g., a room) or stimulus (e.g., a tone or smell). Finally, ablation studies demonstrate that DG adult neurogenesis contributes to cognitive flexibility (Dupret et al., 2008; Garthe et al., 2009; Burghardt et al., 2012), which is the ability to ignore familiar associations when contingencies are changed (Wiskott et al., 2006; Kempermann et al., 2008).

Results obtained from these studies are variable and some can be inconsistent due to such factors as the species and strain used, ages of ablated neurons, methods of ablation, behavioral tasks, and performance analyses used. It is important to note that studies using anti-mitotic drugs or x-irradiation to target adult neurogenesis also affect other proliferating precursor cells and mature cell types, and can also be incomplete (Dupret et al., 2005; Zhang et al., 2008). Ablation of adult-born neurons in the DG also impairs synaptic plasticity, so behavioral deficits are also associated with impaired DG plasticity (Ming and Song, 2005; Massa et al., 2011).

Neurogenic-dependent plasticity is also found in the OB (Gheusi et al., 2000; Enwere et al., 2004; Imayoshi et al., 2008) and ablation of OB neurogenesis impairs spontaneous olfactory behavior, associative olfactory tasks and olfactory-dependent behaviors (Gheusi et al., 2000; Mak et al., 2007; Breton-Provencher et al., 2009; Valley et al., 2009; Sultan et al., 2010; Kageyama et al., 2012). However, numerous studies have, by contrast, demonstrated that ablation or reduction of adult neurogenesis in the OB need not impair odor-discrimination or odor-association ability (Imayoshi et al., 2008; Breton-Provencher et al., 2009; Lazarini et al., 2009; Valley et al., 2009; Sakamoto et al., 2011; Feierstein, 2012), although neurogenesis in this region may still selectively mediate relatively fine-scale odor-pattern separation (Sahay et al., 2011). In support of this role, enhanced discrimination of perceptually-similar odors, decreased odor-discrimination times, and higher responsivity to novel odors are associated with increased survival of newborn olfactory bulb neurons (Mandairon and Linster, 2009; Moreno et al., 2009; Mouret et al., 2009).

Ablation studies demonstrate that adult neurogenesis is essential for the function of the brain structures involved. Abolishing DG neurogenesis results in deficits in hippocampal-dependent memory, and abolishing OB neurogenesis results in deficits in olfactory-dependent behaviors. A key functional overlap between the DG and OB that is suggested by ablation studies is pattern separation, where similar experiences or events are transformed into discrete, non-overlapping representations (Sahay et al., 2011). Discrimination and association of both spatial and olfactory stimuli or cues as measured in several ablation studies are aspects of pattern separation. In support of this hypothesis, ablation studies are able to show stronger behavioral deficits when a more difficult spatial (Drew et al., 2010) or olfactory (Moreno et al., 2009) learning task is employed. Thus, adult neurogenesis in both the DG and OB may be involved more in fine, than large, scales of input discrimination.

## Potential disadvantages of adult neurogenesis

The observation that adult neurogenesis is largely restricted to the DG and OB, and varies in prevalence across vertebrate taxa (Amrein and Lipp, 2009; Barker et al., 2011), suggests that it involves costs, or potential costs, as well as benefits. Some such costs may be more or less fixed and mechanistic, such as the energetics of producing new neurons, whereas other costs would manifest due to the potential for sub-optimal function, and disease, should adult-neurogenesis systems become dysregulated. Such costs have seldom been addressed in the literature relevant to the adaptive significance of adult neurogenesis in mammals, or its restriction to the DG and OB, despite their potentially important roles in mediating variation in neurogenesis patterns among taxa. In this section, we discuss evidence to suggest that dysregulated adult neurogenesis contributes to some neurological disorders and brain tumorigenesis in humans and rodent models. In free-living mammals, the fitness costs of psychological dysfunctions, or brain tumors, are expected to be at least as severe as in humans.

### Psychiatric disorders

In humans, aberrant adult neurogenesis has been linked to epilepsy (Hattiangady et al., 2004; Jessberger et al., 2007), intellectual disability (Luo et al., 2010; Scotto-Lomassese et al., 2011), affective disorders (Samuels and Hen, 2011; Valvezan and Klein, 2012; Petrik et al., 2012; Lee et al., 2013), anxiety disorders (Revest et al., 2009), and, especially, schizophrenia (Reif et al., 2007; Toro and Deakin, 2007; Inta et al., 2011). We focus on schizophrenia, as it has been subject to the most-intensive study, and, importantly, hippocampal and olfactory function are notably compromised in schizophrenic patients, as described below.

The first study to directly link adult neurogenesis to schizophrenia in humans showed that precursor cell proliferation in patients was reduced by 63% (Reif et al., 2006). Interestingly, atypical neuroleptics increase adult neurogenesis (Wakade et al., 2002). Another intriguing link between schizophrenia and adult neurogenesis is the late (post-adolescent) onset of the disorder. Several environmental triggers for psychosis are important modulators of adult neurogenesis. Adult neurogenesis is particularly sensitive to stress (Mirescu and Gould, 2006; Snyder et al., 2011) and drugs of abuse (Eisch et al., 2000; Duman et al., 2001; Yamaguchi et al., 2004), suggesting that dysregulated adult neurogenesis may be a precipitating or contributing factor in schizophrenia pathology. Such inferences are supported by both genetic and behavioral studies.

#### Genetic evidence

Many schizophrenia-risk genes regulate neuronal development and plasticity. Some of these also regulate adult neurogenesis. DISC1 is one of the most studied genes associated with schizophrenia, and mutations in the gene are generally associated with decreased function of the protein. DISC1 modulates neuronal migration and differentiation, neurite outgrowth and synaptic plasticity (Ishizuka et al., 2006). In the adult brain, DISC1 is expressed at high levels in the DG of the hippocampus and at lower levels in the OB (Austin et al., 2004). Importantly, DISC1 mutant mice show deficits in spatial working memory (Koike et al., 2006; Kvajo et al., 2008; Pletnikov et al., 2008) and olfaction (Hikida et al., 2007). Duan et al. (2007) first demonstrated that DISC1 regulates the temporal maturation and integration of new neurons in the DG during adult neurogenesis. Mutation or downregulation of DISC1 accelerates neuronal integration and also leads to aberrant positioning, morphology, and plasticity of newborn cells (Duan et al., 2007; Mao et al., 2009; Kvajo et al., 2011). DISC1 inactivates GSK3-beta signaling, another key regulator of adult neurogenesis implicated in both schizophrenia and bipolar disorder (Brandon et al., 2009). Another well-characterized gene, Reelin, is consistently downregulated in schizophrenia and is important for regulating neuronal migration and integration during early postnatal neurogenesis. Similar to DISC1, downregulation of Reelin signaling in adult neuron progenitors also leads to aberrant migration and integration of new neurons in the adult DG (Teixeira et al., 2012). Another schizophrenia risk gene, neuregulin-1, regulates the identity of proliferating precursor cells (Ghashghaei et al., 2006). Knockout mice for the transcription factor Npas2 demonstrate behavioral and neuroanatomical phenotypes consistent with schizophrenia (Erbel-Sieler et al., 2004) and a significant reduction in adult neurogenesis might explain the abnormalities seen in these mice (Pieper et al., 2005).

Given that schizophrenia is a neurodevelopmental disorder, the degree to which schizophrenia risk genes mediate disease though effects on adult neurogenesis, compared to, or in conjunction with, effects on neurogenesis in early development, remains unclear. Presumably, these two temporally-distinct arenas for neurogenesis should be genetically dissociable to some degree, which would allow partitioning of early vs. adult effects. In any case, studies of schizophrenia provide evidence that liability to this severe disorder is mediated in part by genetically-based alterations to neurogenesis.

#### Behavioral evidence

Behavioral studies in schizophrenic patients and mouse models of schizophrenia highlight several similarities between schizophrenia phenotypes and cognitive deficits following loss of adult neurogenesis (Eriksson, 2006; Eisch et al., 2008; Kvajo et al., 2008; Balu and Lucki, 2009). Adult neurogenesis is thus required for normal hippocampal function, and hippocampal function appears to be especially-highly compromised (compared to other brain regions) in schizophrenia (Harrison and Eastwood, 2001; Harrison, 2004). This brain region also shows the largest volumetric loss in schizophrenia (Arnold, 2000; Hemby et al., 2002). In the DG, adult neurogenesis mediates the correct encoding of new memories in order to decrease interference between memories (pattern separation, as described in more detail below) and to separate information based on spatial and temporal characteristics (Deng et al., 2010; Koehl and Abrous, 2011). Memory deficits in schizophrenia patients directly involve such encoding errors, rather than errors in storage or retrieval (Talamini et al., 2010). Schizophrenia patients also perform poorly on tasks requiring integration of object and spatial information (Waters et al., 2004; Talamini et al., 2005; Kravariti et al., 2006; Talamini and Meeter, 2009), and have difficulties binding different features of events (such as content and context) into complete representations (reviewed in Mitchell and Johnson, 2009). These impairments have been suggested to arise from reduced cortical connectivity upstream of hippocampal inputs (Harrison and Eastwood, 2001), but they also fit with a hypothesis of reduced or dysregulated adult neurogenesis.

Reports of olfactory deficits in schizophrenia patients fit clearly with the framework that adult neurogenesis is dyregulated in schizophrenia. Several studies over the past two decades have found that patients with schizophrenia have reduced olfactory function (Kopala et al., 1993; Moberg et al., 1997, 1999). Schizophrenia patients have reduced olfactory discrimination ability (Austin et al., 2004; Tueting et al., 2006) and impaired detection of odor dimensionality (Atanasova et al., 2008). Taken together, schizophrenia thus comprises several behaviors that are consistent with decreased or dysregulated adult neurogenesis.

Selective pressures related to cognitive-behavioral dysfunction should be especially important in highly-social, large-brained species such as humans and other primates, which may help to explain the low rates of neurogenesis within this group. Among mammals other than humans, effects of dysregulated neurogenesis on cognition would presumably exert deleterious effects in the context of degraded behavioral and ecological functions. Such effects should be important to study, as they are highly relevant to the selective maintenance of neurogenesis and its restriction to the DG and OB. Ablation studies that involved behavioral assays with higher and more direct levels of ecological validity (i. e., resemblance to social behavior typical of rodents in the wild) should be especially useful in this regard (e.g., Lévy et al., 2011).

### Brain tumor risk

Brain cancer has one of the lowest incidences for cancer, but one of the worst prognoses (Deorah et al., 2006; Kohler et al., 2011). Astrocytes and oligodendrocytes were once thought to be the only dividing cells in the postnatal brain that were susceptible to transformation. Neural stem cells in the adult brain are now the most likely candidates in the adult brain to undergo aberrant genetic and epigenetic changes into tumor-forming stem cells (Dirks, 2008; Harris et al., 2008; Kroonen et al., 2011). Other candidate tumor-forming stem cells include neuroepithelial cells, transient amplifying precursors in the adult SVZ and oligodendrocyte progenitor cells in white matter (Sanai and Alvarez-Buylla, 2009). The SVZ in particular is believed to be an important source of malignant brain tumors (Sanai et al., 2005; Vescovi et al., 2006). NSCs undergo self-renewal and are therefore susceptible to mutation and dysregulated proliferation and differentiation that could increase risk for tumor formation. If mechanistic dysregulation of adult neurogenesis contributes to the etiology of brain tumors, there are two main predictions: (1) that common genes and signaling pathways regulate both adult neurogenesis and tumorigenesis, and (2) that brain tumors will initiate or cluster near the two neurogenic regions of the brain. To the extent that brain cancer risk is associated with adult neurogenesis, it may help to explain why neurogenesis is restricted to the DG and OB, and why its importance varies among mammal species that differ in such variables as lifespan and brain size.

#### Common genes and signaling pathways

Cancer stem cells (CSCs) have been isolated from major malignant brain tumors and found to share key properties of adult neural stem cells (NSCs) (Ignatova et al., 2002; Singh et al., 2003; Galli et al., 2004). CNCs and NSCs also share similar perivascular niches (Calabrese et al., 2007). Common transcription factors regulate both adult neurogenesis and tumorigenesis. Many transcription factors involved specifically in neural fate specification and differentiation are dysregulated in various types of brain tumor cells (Hide et al., 2009; Elsir et al., 2010; Huang et al., 2011; Hsieh, 2012; Kaminska et al., 2013). For example, the transcription factor TLX is required for glioblastoma formation in the adult neurogenic niche (Zou et al., 2012). TLX is expressed exclusively in NSCs and positively regulates self-renewal, maintenance and migration of neural precursors (Liu et al., 2010). Overexpression of TLX increases cell division in the SVZ, and also stimulates angiogenesis (Liu et al., 2010). Tumor suppressive pathways are also major regulators of adult neurogenesis (Zhao et al., 2008; Hsieh, 2012; Zou et al., 2012; Bartesaghi and Salomoni, 2013). PTEN is an important tumor suppressor gene regulating neuronal differentiation (Lachyankar et al., 2000), precursor migration (Li et al., 2003) and also tumor initiation and maintenance (Hongwu et al., 2008). Deletion of cell-cycle inhibitors such as p16, p21, and p53 leads to increased proliferation of neural progenitors (Ming and Song, 2011). Conversely, increased expression of these cell-cycle inhibitors correlates with decreased progenitor proliferation (Molofsky et al., 2006). Interestingly, expression of some cell-cycle inhibitors has been found to be much higher in the rostral migratory stream (RMS) and DG of adult brains than in other regions (Medrano and Scrable, 2005; Meletis et al., 2006). Unique cytoskeletal proteins, growth factors, and telomerase expression patterns are also similar in neurogenic cell populations and brain tumor cells (Sanai and Alvarez-Buylla, 2009).

**Figure 4:**
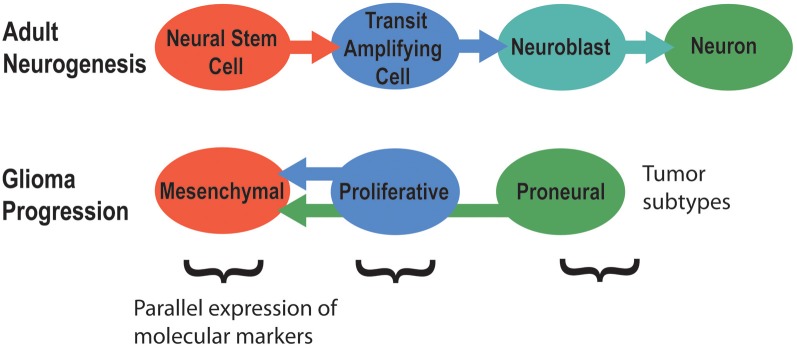
**The molecular signatures of tumor subtypes parallel the stages of adult neurogenesis**. Adapted from Phillips et al. (2006).

Signaling pathways that regulate adult neurogenesis can mediate brain tumor formation and also promote tumor aggressiveness (Phillips et al., 2006). Tumors of poor prognosis such as grade IV glioblastomas generally express markers of neural stem cells, while grade III tumors usually express markers of neuroblasts or neurons. Hence, brain tumors might originate from different stages of adult neurogenesis (Singh et al., 2003; Phillips et al., 2006; Rahman et al., 2009; Yadirgi and Marino, 2009; Bartesaghi and Salomoni, 2013). The primary model for this idea is that increasing stages of glioma progression match earlier stages of adult neurogenesis in terms of cell markers, morphology, and activated signaling pathways (Figure 4) (Phillips et al., 2006). Hence, NSCs of the subventricular zone (SVZ) are suspected to be the origin of glioblastoma brain tumors, the most common and malignant of brain cancers (Galli et al., 2004; Liu et al., 2010; Sanai, 2010; Kohler et al., 2011). Differentiated cells in the brain can also undergo dedifferentiation to generate a NSC or neural progenitor that then initiates and maintains tumor progression (Friedmann-Morvinski et al., 2012). Overall, these studies demonstrate how dysregulated mechanisms of signaling in adult neurogenesis might contribute to brain tumor formation.

#### Regional distribution of tumors in the brain

There is little clinical data available regarding specific regionalization of tumors to neurogenic zones. However, there is some evidence to suggest that the SVZ environment in particular is involved in brain tumor formation, likely via signaling pathways that are largely expressed in this area, as described above. Thus, many types of brain tumors most frequently arise in brain regions adjacent to the SVZ (Sanai, 2010), and in particular, many gliomas are periventricular or contiguous with the SVZ (Lim et al., 2007). Mice lacking certain tumor suppressor genes in the CNS first develop tumors in the SVZ (Zhu et al., 2005). Regions of the brain with proliferative cell populations such as the SVZ are also more sensitive to chemical or viral transformation and tumor formation (Sanai and Alvarez-Buylla, 2009). Transformed neural precursors can migrate along white matter tracts and blood vessels, making it difficult to determine their area of origin (Zhu et al., 2005; Calabrese et al., 2007). Migration of transformed neural stem cells and their precursors may explain why gliomas later lose evidence of being contiguous with the SVZ (Sanai and Alvarez-Buylla, 2009). Human glioblastoma cells injected into the striatum of immunodeficient mice migrate specifically toward neurogenic zones (e.g., the SVZ); these cancer cells then take on NSC markers and migrate preferentially to the OB (Kroonen et al., 2011). An interesting hypothesis arising from this study is that neurogenic niches might provide a substrate for secondary tumor formation or recurrence (Goffart et al., 2012). These results further suggest that maintenance of neural stem cell populations in the SVZ may be an important risk factor for brain tumorigenesis. While these neural stem cells are important for adult neurogenesis, other cell types, such as astrocytes and oligodendrocytes, are also generated from these stem cells in the adult brain and migrate to different regions of the brain (Shen et al., 2004).

Direct links of neural stem cells in the DG, and especially the OB, with tumor formation have yet to be established. However, to the extent that brain tumor risk represents a selective factor mediating the taxonomic distribution of neurogenesis, it may help to explain the especially-low rates of neurogenesis in bats and humans, because both of these taxa exhibit relatively long lifespans (Amrein et al., 2007) that should notably potentiate or increase the prevalence of cancer.

## Hypotheses for the adaptive significance of adult neurogenesis

Most studies of adult neurogenesis have centered on the DG, even though olfactory-system adult neurogenesis appears to be more-widespread across animals (Barker et al., 2011). The role of neurogenesis in olfaction has thus remained largely obscure (Sakamoto et al., 2011). Three non-exclusive hypotheses have, however, recently been proposed to explain the significance of adult neurogenesis exclusively or mainly in the mammalian DG and OB. The first two hypotheses focus primarily on the proximate mechanisms of adult neurogenesis, but have direct implications for the perceptual, ecological and life-historical situations that should favor or disfavor adult neurogenesis. By contrast, the third hypothesis specifies particular environmental circumstances that promote neurogenesis, but also has implications for understanding proximate mechanisms.

**Figure 5:**
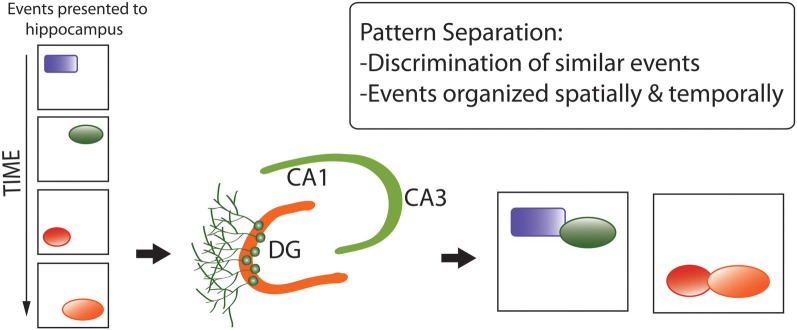
**Pattern separation hypothesis of adult neurogenesis, developed for the dentate gyrus but recently extended to the olfactory bulb**. Pattern separation refers to the discrimination between similar, complex sets of environmental stimuli (e.g., spatial information) for the generation of representations in memory (Aimone et al., 2010). Here, “events” refer to components of memories that are very similar and easily confused for one another. (e.g., similar spatial cues in a similar environment). Pattern separation enables these components of memory to be encoded into distinct complex memory representations that are unique and less easily confused. They also retain their temporal structure which is largely provided by having immature neurons in the DG circuitry.

First, the *pattern separation* hypothesis (Figure 5) proposes that adult neurogenesis serves to generate neuronal networks better-capable of discriminating between similar, complex sets of environmental stimuli for generation of representations in memory (Clelland et al., 2009; Aimone et al., 2011; Sahay et al., 2011; Kesner, 2013). This hypothesis was originally proposed in the context of neurogenesis in the DG (reviewed in Yassa and Stark, 2011), and extended to OB neurogenesis by Wilson (2009) and Sahay et al. (2011) under the supposition of analogous functions for spatial-contextual discrimination by the hippocampus and odor discrimination by the olfactory system, both centrally involving pattern separation and completion.

Wilson's (2009) and Sahay et al.'s (2011) extensions of the pattern-separation hypothesis to olfaction are consistent with a variety of findings, such as decorrelation of highly-similar complex odorant mixtures by rat OB neurons (Barnes et al., 2008), increased survival of new adult-generated OB neurons under odor enrichment conditions (Rochefort et al., 2002; Barker et al., 2011; Nissant and Pallotto, 2011, for reviews), and deficits in odor perception and discrimination as demonstrated by ablation studies (Moreno et al., 2009; Mouret et al., 2009). A more-general caveat regarding the pattern separation hypothesis is that this neuronal-population function is not necessarily unique to the DG (and the OB), such that the hypothesis cannot necessarily explain why neurogenesis is largely restricted to these two regions. This hypothesis predicts that neurogenesis is favored in circumstances and species where fitness is enhanced via discrimination between similar, complex sets of environmental stimuli, to generate useful representations in memory.

**Figure 6:**
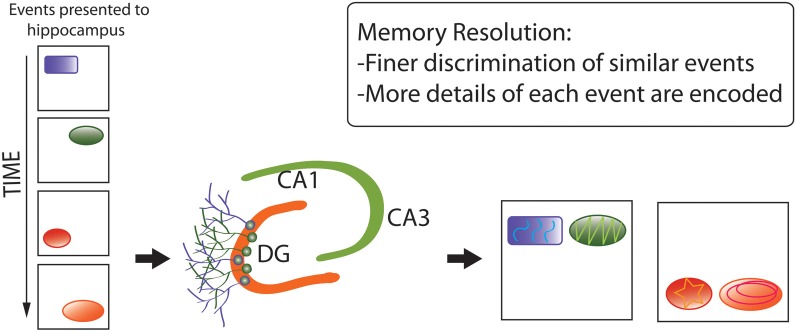
**Memory resolution hypothesis of adult neurogenesis**. Whereas pattern separation represents an effect or component of the primary, over-arching function of adult neurogenesis, memory resolution increases the amount and structure of information encoded by particular brain regions via a combination of (1) younger neuronal populations with low-specificity, densely-sampled representations, which better-encode novel environmental features, with (2) older neurons with high-specificity, sparsely-sampled representations, encoding more-familiar aspects of the environment. The diagram again shows that distinct, complex memory representations are encoded, but also that they are more separated from each other and more detail is encoded.

Second, the *memory resolution* hypothesis (Figure 6) (Aimone et al., 2011) posits that pattern separation represents an effect or component of the primary, over-arching function of adult neurogenesis: increasing the amount and structure of information encoded by particular brain regions, via combination of (1) young neuronal populations with low-specificity, densely-sampled representations of inputs, which better-encode novel environmental features, with (2) older neurons with high-specificity, sparsely-sampled representations, encoding more-familiar aspects of the environment (Marín-Burgin et al., 2012). By this hypothesis, mixed populations of young and mature neurons can dynamically optimize levels of memory resolution as functions of environmental novelty (and possibly other factors, such as fine-scale complexity), thereby adapting neuronal-network functions to changing sensory demands.

The memory resolution hypothesis was developed in the context of hippocampal neurogenesis, but should, in theory, be equally applicable to other neurogenic regions, especially the OB. The hypothesis is supported by findings such as differential impairment of finer-scale discrimination under ablated neurogenesis (see, e.g., Kesner, 2013), but robust tests require experimental separation of encoding from retrieval functions, in relation to familiarity vs. novelty of the environment, under different levels of neurogenic activity (Aimone et al., 2011). According to this hypothesis, adult neurogenesis should be expected in species and situations where encoding of novel, dynamic, extensive, and complex information are at a selective premium.

**Figure 7:**
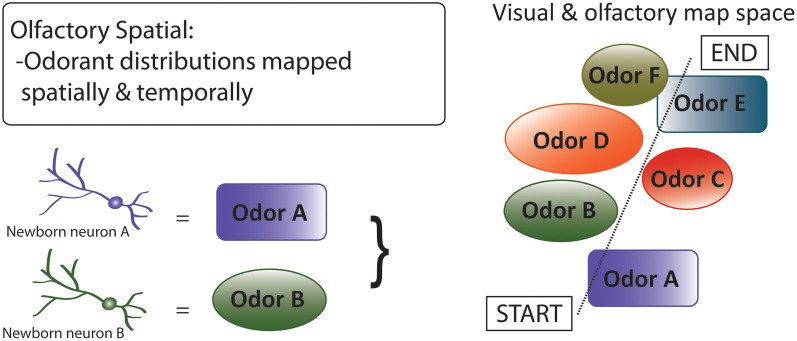
**Olfactory spatial hypothesis of adult neurogenesis**. By this hypothesis, adult neurogenesis subserves spatial-navigational functions by functionally decoding and mapping odorants (Jacobs, 2012). The hypothesis focuses on the central roles of high-resolution spatially-explicit olfaction in mammalian food location, individual recognition, homing, and anti-predator behavior. Diagram shows a “complex spatial” environment with various odors that are represented or encoded by adult born neurons, which then represent the visual and olfactory map space. This hypothesis can be extended to DG function, where other contextual information (e.g., emotional) can be spatially mapped.

Third, by the *olfactory spatial* hypothesis (Figure 7), neurogenesis in the OB and DG predominantly subserves spatial-navigational functions, via an integrated system that includes decoding and mapping of odorants (Jacobs, 2012). By this hypothesis, adult neurogenesis in the DG involves encoding of a bearing map that depends on directional cues, such as olfactory plumes, which require locomotion to be detected (Jacobs and Schenk, 2003); the hypothesis thus explains restriction of adult neurogenesis to this specific region of the hippocampus. With regard to olfaction, the hypothesis focuses on the central roles of high-resolution spatially-explicit olfaction in mammalian food location, homing, anti-predator behavior, and social interactions, in the contexts of early mammalian brain expansions dominated by OB enlargement (Rowe et al., 2011), independent, non-allometric scaling of OB size among mammals (Reep et al., 2007), and comparative associations of OB and hippocampal sizes with correlates of spatial-ecological selective pressures (Barton, 2006; Jacobs, 2012). With regard to proximate mechanisms, the hypothesis suggests that neurogenesis in the DG and OB are integrated physiologically and neurologically, at least in mammal species that rely substantially on olfaction.

The olfactory spatial hypothesis is concordant with extensive evidence for olfactory-based spatial learning in laboratory rats and mice (Wiedenmayer et al., 2000; Wallace et al., 2002; Rossier and Schenk, 2003; Porter et al., 2005; Kulvicius et al., 2008) and tight functional coupling of the OB with the DG (Vanderwolf, 2001; Martin et al., 2007; Arisi et al., 2012), but few studies have investigated links between neurogenesis and fine-scale spatial performance mediated by olfaction, or tested for odor discrimination learning in spatial contexts. Most generally, the olfactory spatial hypothesis predicts that integrated adult DG and OB neurogenesis have been optimized, in species that depend on olfaction, for performance of spatial-navigational functions. In contrast to the pattern separation and memory resolution hypotheses, which posit more-general benefits to adult neurogenesis, this hypothesis may help to explain why adult neurogenesis is mainly confined to the DG and OB. Given that species and higher taxa vary considerably in their use of olfactory social and environmental cues, and that use of olfaction may be inversely related to use of high-resolution visual perception (Swaney and Keverne, 2009), the olfactory spatial hypothesis should apply more or less strongly across different taxonomic groups; for example, less strongly in humans (Macklis, 2012) and diurnal primates, and more strongly in nocturnal primate groups and most rodents, which are also nocturnal and rely substantially on spatial navigation, and olfaction, in natural ecological contexts.

**Figure 8:**
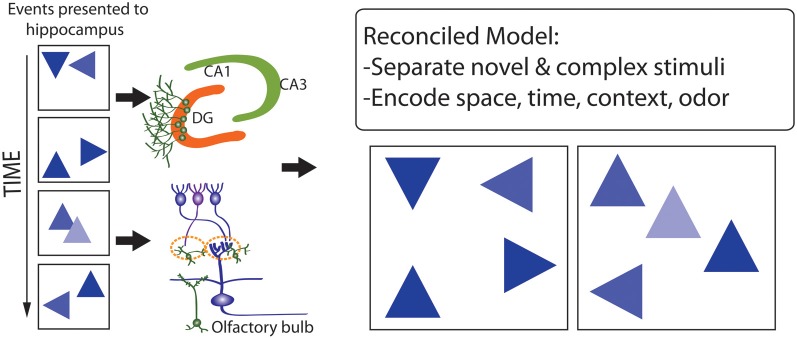
**Model that reconciles the pattern separation, memory resolution, and olfactory spatial hypothesis for adult neurogenesis**. A complex series of events occurs in space and time (**Left**) which are encoded by the DG and OB where they are separated (**Right**, where events or stimuli are more distinguished from one another). Stimuli that are especially novel, complex, and fine-grained, and include spatial-contextual and spatial-odor information, are better processed via adult neurogenesis.

The observation that the pattern separation, memory resolution, and olfactory spatial hypotheses are not mutually exclusive, and differentially emphasize either proximate mechanisms (the former two hypotheses) or ultimate functions (the latter hypothesis), suggests that they may be reconciled, to develop conceptual models (e.g., Figure 8) that help to explain why adult neurogenesis is restricted to roles in processing particular forms of input, but not others. Previous studies have focused on the importance of stimulus novelty and complexity (Aimone et al., 2011; Barker et al., 2011) in this context, such that spatial-contextual information processing, and processing of chemical odors (linked with spatial representations), may involve higher levels of stimulus dimensional complexity, and more-frequent stimulus novelty, than processing of dimensionally-simpler and more temporally-stable visual-object, visual-location, auditory, and tactile stimuli. Higher levels of stimulus novelty in particular may underlie advantages of adult neurogenesis, in comparison with synaptic plasticity, for stimulus processing and memory formation where natural selection has favored delicate balancing of representation stability with flexibility, under conditions where new inputs provide information highly salient to optimizing behavior, learning and memory.

This hypothesis described above uniquely predicts that spatial and odorant complexity and novelty, and selection for fine-scale discrimination abilities, strongly mediate the functional significance of adult neurogenesis in both the DG and OB. How well is this prediction met? First, many of the relatively well-documented correlates of adult neurogenesis in the DG, including younger age, environmental enrichment, and higher physical activity (which normally entails movement in space) (e.g., review in Arisi et al., 2012), are also expected to be associated with higher stimulus novelty.

Second, adult neurogenesis has been linked with higher task difficulty in discrimination, relatively fine-scale odor discrimination, and odor novelty (rather than just enrichment *per se*) (Veyrac et al., 2009; Alonso et al., 2012; Lemaire et al., 2012), all suggesting key roles for differential survival and function of younger neurons in stimulus novelty and complexity. Third, novel odors are indeed responded to preferentially by younger granule cells (Magavi et al., 2005; Belnoue et al., 2011), and several emerging, strong correlates of olfactory-system neurogenesis include recognition of new conspecifics including newborn pups or adult social interactants, which represent notably-novel, and complex, olfactory stimuli (Lévy et al., 2011; Kageyama et al., 2012). This hypothesis also generally fits with the developmental, social, and life-historical patterns and trajectories of neurogenesis, and much of the among-species comparative data (e.g., Barker et al., 2011; Feierstein, 2012), and experimental data that implicates DG neurogenesis in spatial context and learning (Kesner, 2013). Additional theoretical-computational studies, and experimental and comparative tests that focus specifically on stimulus novelty and complexity, and spatial-context effects, are necessary to evaluate these ideas further. In particular, the relative importance, and inter-relationships, of stimulus complexity, novelty, and fine scale require focussed experimental studies.

## Comparative tests on the adaptive significance of adult neurogenesis in hippocampus and olfactory bulb

Most comparative tests for the adaptive significance of brain size and structural variation of brain components in vertebrates have centered on neocortex size in relation to social group size and other socioecological traits in primates and other mammalian taxa (e.g., Dunbar, 1998), and hippocampus size with regard to ecological factors that select for enhanced spatial learning and memory, such as food caching in birds (e.g., Lucas et al., 2004). To comparatively evaluate the hypotheses that adult neurogenesis has been maintained predominantly under spatial selective pressures using currently-available data, it is necessary to use hippocampus size as a proxy for size of the DG (and a stronger role for adult neurogenesis), and OB size as an indicator of adult neurogenesis in this region. This strong assumption is predicated on an integral, fundamental role for adult neurogenesis in overall hippocampal and OB functioning, such that larger size of these brain regions is positively associated with the importance of adult neurogenesis in some taxa, compared to others. The assumption is supported by studies of birds that demonstrate positive covariation of hippocampus size with rates of neurogenesis, both across and within species (Sherry and Hoshooley, 2010; Barnea and Pravosudov, 2011), but it requires testing in primates once more data are available. In any case, if the sizes of neither the DG nor OB is associated with among-species variation in spatial-ecological or spatial-social traits in the primate species considered below, then it is relatively unlikely that such factors mediate variation in their central neurological functions such as adult neurogenesis.

For primates, two previous studies have investigated the social and ecological correlates of OB size, or hippocampus size. Barton (2006) tested for ecological factors linked with OB size in the two main lineages of primates, haplorhines (most of which are diurnal and gregarious) and strepsirhines (most of which are nocturnal and solitary-territorial). He reported that the relative size of the main OB was larger among strepsirhines than haplorhines, larger in nocturnal than diurnal lineages among the primates investigated, and larger in frugivores + insectivores (which rely on spatially-dispersed foods) than in folivores. Barton et al. (1995) also reported that among nocturnal strepsirhines, the proportion of fruit in the diet was positively associated with relative OB size. These results support the hypotheses of a role for the OB in spatial-environmental tasks, especially among nocturnal species for which strong visual cues are less-notably available.

Edler (2007) tested for ecological correlates of hippocampus size among primates, without differentiating between strepsirhines and haplorhines. She found that a relatively-larger hippocampus (in relation to medulla size) was associated with a more-frugivorous diet, larger home range sizes, and nocturnality, though with some variation from these results dependent upon taxa included and statistical methods used to adjust for overall brain size and phylogenetic structure. Despite such caveats, these findings suggest that primate hippocampus size subserves ecological functions related to spatial selective pressures, as in various other vertebrate taxa (Sherry et al., 1992).

We conducted a comparative study of primate brain regions that centered on the differences between gregarious primates (mainly diurnal haplorhines) and so-called solitary primates (mainly nocturnal strepsirhines). This division was based on the marked differences in social-ecological systems between these two sets of species. Thus, gregarious primate species exhibit complex social systems in which individuals closely, visually attend to the status, social roles and activities, and spatial positions of group members. By contrast, the social system of nocturnal, solitary primates is typically characterized by the existence of a mosaic of fully or partially-overlapping individual territories which are scent-marked and defended, with spatially-dispersed monogamy or multimale reproductive systems (Bearder, 1987, 1999). In such systems, the size of territories and the degree of their overlap between and within sexes is a primary determinant of mating system and interaction frequency between individuals. The system has been termed “socio-territoriality” (Génin, 2010) in which the relevant social units are spatial groups of overlapping home ranges within which interactions are friendly, in contrast with unfriendly interactions across non-overlapping groups (Wiens and Zitzmann, 2003).

We postulated that in solitary primates the main form of social-ecological information is concerned with memory for the positions of individuals and resources, and territorial boundaries, within a spatial landscape structured by both dimensional position and social identities of interacting individuals as determined by scent. As such, both hippocampus and OB sizes should be associated with measures of spatial selective pressures among solitary primates, although not necessarily among gregarious primates, for which social, sexual and ecological interactions are mediated predominantly by ongoing visual input in contexts not directly-reliant on spatially-explicit memory.

We tested this hypothesis in a multivariate study of the relationship between two socio-ecological spatial life history variables, population density and home range size, and brain component volumes in primates grouped according to whether they are solitary or gregarious. For solitary species, we regard population density as an indicator of interaction frequency, and home range size functions as a proxy for the difficulty with which individuals are tracked across a territory and the spatial complexity of a territory in general. Data on primate social group size, population density and home range size were obtained from a comprehensive curated database of mammalian life history (Jones et al., 2009). Data on brain component volumes in 44 species (35 gregarious, 9 solitary) were gathered from the literature (**Table S1**), and all variables were log transformed prior to analysis.

Structural equation models, which allow the construction of statistical models incorporating quantitative data and their associated measurement errors into a theoretically informed causal framework, were used to analyze the data, using IBM Amos software. The models were based on a hierarchical allometric approach in which each physical brain component is predicted on the basis of the higher-order component of which it is a part. For example, the volume of the medial amygdala is predicted by the volume of the whole amygdala, which in turn is predicted by the volume of the telencephalon, which in turn is predicted by the volume of the whole brain. The highest-order predictor was body mass. Associations of the socio-spatial variables with the brain allometry model were assessed by identifying significant pathways between these variables and brain component volumes, and minimizing the number of parameters in the final model, by moving predictive pathways up the allometric hierarchy when justified by AIC score.

Statistical analyses of species data are commonly subject to transformations that adjust for phylogenetic structure. These transformations are not well-suited to cases in which the evolution of multivariate continuous characters depend on the evolution of an independent discrete character (such as gregarious vs. solitary) whose ancestral states are not known with certainty. We accounted at least in part for phylogenetic structure of the data by first fitting the brain allometry model to all species and permitting independent intercepts for each component for haplorhines vs. strepsirhines. These intercepts were then fixed for further analyses which divided the taxa into solitary vs. gregarious groups, such that the allometric effects of being a strepsirhine or haplorhine are independently, as fixed constants, built into the model. The significance of differences between gregarious and solitary groups were assessed using chi-square tests.

**Figure 9:**
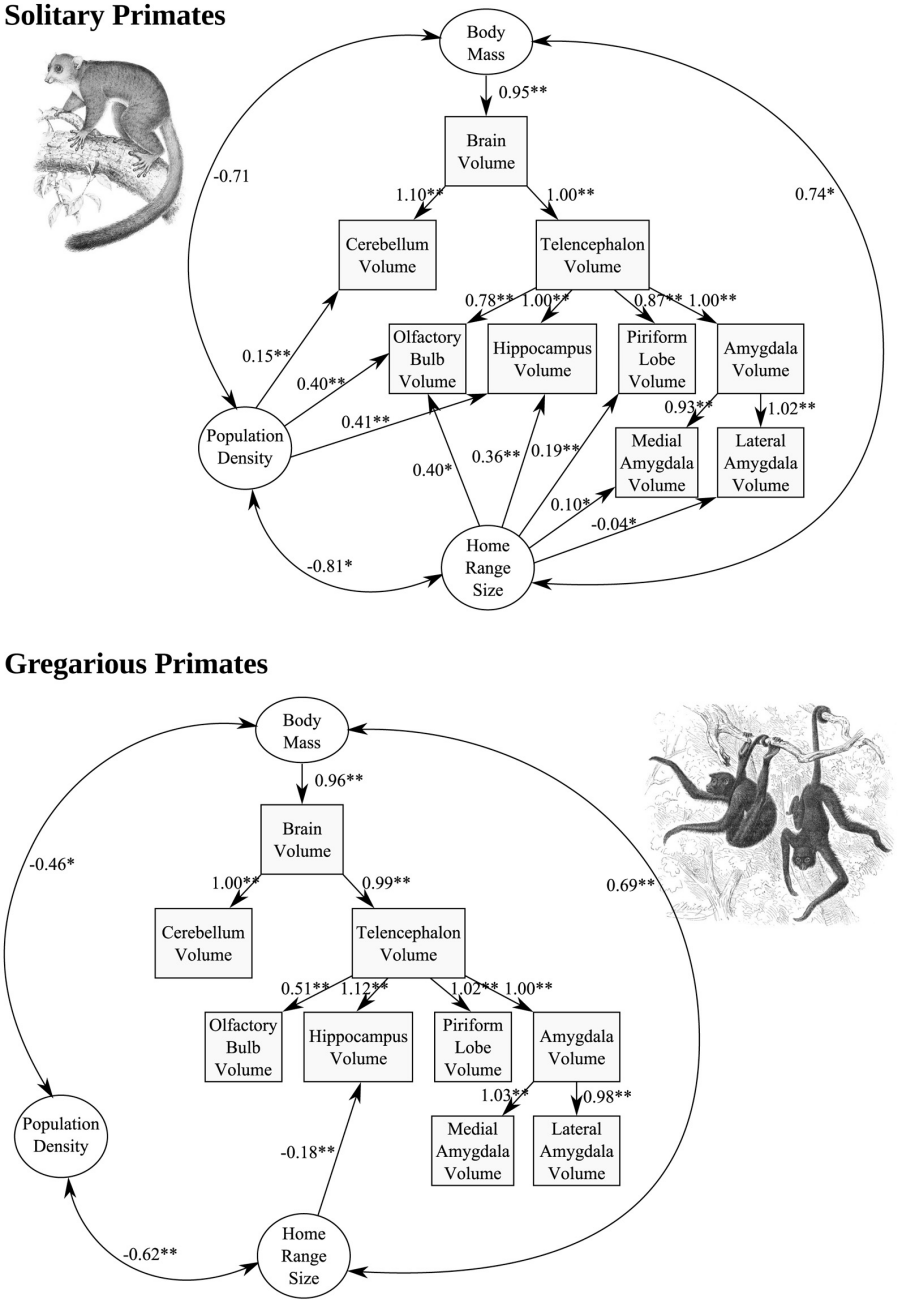
**Structural equation models of the effect of population density and home range size, two variables of importance to the territorial social system of solitary primates, on the allometry of brain components**. Each standardized path coefficient indicates the number of standard deviations increase in the dependent variable caused by an increase of one standard deviation in the independent variable; coefficients are significant at α = 0.01 (two asterisks) or α = 0.05 (single asterisk).

In solitary species (but not in gregarious species), population density exerted a significant positive effect on OB (*p* = 0.009), hippocampus (*p* < 0.001), and cerebellum (*p* < 0.001), volume, while home range size was found to exert a significant positive effect on OB (*p* = 0.012), hippocampus (*p* < 0.001), and piriform lobe (*p* < 0.001) volume (Figure 9). Chi-square tests indicated that all of these relationships differ significantly from those found in gregarious species at α = 0.01. The only significant path coefficient found in gregarious taxa was a negative correlation between home range size and hippocampus volume (*p* = 0.002).

The generality and robustness of these results is limited by the small number of solitary species for which brain and ecological data are available (*N* = 9), and the need for additional, larger-scale phylogeny-based analyses, once more data are available. However, the larger hippocampus, OB, and piriform lobe, in solitary but not gregarious primate species with high population densities, larger home range sizes, or both, supports the hypothesis that these brain regions are involved in processing of spatially-structured information, presumably including both position itself (with regard to territory locations) and socio-spatial olfactory-based identities of conspecifics, identified through scent-marking. Anatomically, the OB projects directly to the piriform cortex, as well as to the entorhinal cortex and amygdala (Mouly and Di Scala, 2006); this system, and its links with the hippocampus (e.g., Arisi et al., 2012), appear uniquely suited for the cognitive demands imposed by the social and physical environment of nocturnal primates, which involve complex spatial processing in three dimensions, coupled with recognition and learning of olfactory social identities and their social salience. The restriction of the relationships observed to solitary, nocturnal primate species is consistent with the negative associations between the sizes of visual compared to olfactory brain structures across primates as a whole, generated predominantly by the differences between diurnal and nocturnal species (Barton et al., 1995; Smith et al., 2007), which largely coincides with haplorhines compared to strepsirhines. The negative association between home range size and hippocampus volume found only in gregarious taxa represents an intriguing result, which suggests that socio-ecological selective pressures on hippocampus size may differ profoundly between gregarious and solitary primate lineages. Among other taxa (mainly birds and rodents), positive comparative associations between home range sizes and hippocampus sizes have been reported (e.g., Healy and Hurly, 2004; Jacobs and Spencer, 2008; Yaskin, 2011), again suggesting that gregarious primates differ in some important regard, perhaps related to their high degree of sociality.

The degree to which reliance on, or contextual uses of, adult neurogenesis in the hippocampal DG and OB of primates and other mammals differ between taxa with diurnal vs. nocturnal activity patterns remains unclear, but OB neurogenesis appears highly limited in (diurnal) humans compared to other mammals thus far investigated (Bergmann et al., 2012), and nocturnal primates may resemble nocturnal rodents (such as rats and mice) more than diurnal primates with regard to their spatial ecologies. Our results do, however, fit with the general hypothesis that restriction of adult neurogenesis to the DG and OB may be mediated by complex, fundamentally spatial-information processing and storage functions subserved exclusively or predominantly by these two regions of the brain, as predicted by a model that reconciles the pattern separation, memory resolution, and olfactory spatial hypotheses, described above.

Future comparative studies with larger samples, more-explicitly phylogenetically-structured analyses, and experimental studies of mammalian neurogenesis in relation to taxonomic variation in spatial and sensory ecology, will be required to evaluate these hypotheses in greater detail. However, our results suggest possible productive avenues for integration of theoretical models of neurogenesis with empirical work, in particular by focusing on comparisons of related nocturnal vs. diurnal mammalian groups. Our results suggest in particular that nocturnal strepsirhines should demonstrate more-developed systems of adult neurogenesis than do haplorhines.

## Conclusions

We have provided an overview of the mechanisms and adaptive significance of adult neurogenesis in mammals focused on the question of why this process is largely restricted to two brain regions, the DG and OB that otherwise appear unrelated in their functions and forms of information processing. A primary conclusion is that the benefits of adult neurogenesis in the DG and OB appear to involve encoding and decoding of relatively novel and complex information that commonly includes spatial components, and that such benefits appear to rely on the properties of relatively-young neurons. The main usefulness of this hypothesis is that it can conceptually unify the proposed information-processing contexts of adult neurogenesis, it is concordant with results from the bulk of ablation and computational studies, and it also fits with many comparative results, including those presented here. An important limitation of the hypothesis is that it has yet to be evaluated directly using experimental or comparative data. Moreover, although neurogenesis appears to be associated with notable potential costs, these costs have yet to be quantified in ecologically-valid contexts, and so remain largely conjectural. As a result, the role of costs in restriction of adult neurogenesis to the DG and OB, and variation among taxa in its overall prevalence, remains unclear. Future studies that integrate benefits with costs, in the context of testing specific alternative models, will be required to further elucidate the adaptive significance of adult neurogenesis.

### Conflict of interest statement

The authors declare that the research was conducted in the absence of any commercial or financial relationships that could be construed as a potential conflict of interest.
